# Update on the Pathophysiology, Aetiology and Management of Central Sleep Apnoea

**DOI:** 10.1002/resp.70286

**Published:** 2026-07-24

**Authors:** Sahan D. Chandrasekara, Matthew T. Naughton

**Affiliations:** ^1^ Department of Respiratory Medicine Alfred Hospital Melbourne Victoria Australia; ^2^ School of Translational Medicine Monash University Melbourne Victoria Australia

**Keywords:** central sleep apnoea, heart failure, periodic breathing

## Abstract

Central sleep apnoea (CSA) of the periodic breathing type occurs during the transition between wakefulness and stage 1 and 2 non‐REM sleep. In adults at sea level, it occurs most commonly due to advanced heart failure (HF) or chronic opioid use and in ~5% of obstructive sleep apnoea patients initiating CPAP. The sudden development of CSA in a chronic CPAP user for obstructive sleep apnoea (OSA) may indicate the development of atrial fibrillation. Less commonly, idiopathic CSA occurs in which cardiac, neurological and endocrine causes should be excluded. Each cause of CSA has a characteristic appearance of apnoea, hyperpnoea and cycle length. Altitude induced CSA has recently been shown to reduce work of breathing whilst maintaining gas exchange, compared with regular breathing and thereby be considered adaptive rather than detrimental. The management of each type of CSA should be directed at the cause. Where there is residual CSA (e.g., post intensive treatment for HF), debate exists as to the next option. One argument is that CSA is adaptive (as seen with altitude) and therapeutic. An alternative argument is to treat the CSA directly with therapies such as respiratory stimulants (e.g., acetazolamide), positive airway pressure (e.g., adaptive servo‐ventilation (ASV)), supplemental oxygen or phrenic nerve pacing. These four latter treatments remain speculative without strong foundations for recommending especially ASV in HF with reduced ejection fraction where increased mortality has been observed.

## 1 Introduction

The term CSA refers to an oscillatory breathing pattern with hyperventilation (lasting < 90 s) followed by an absence of ventilation (lasting < 90 s) and a total cycle length (combined hyperventilation and apnoea lengths) < 3 min [1, 2]. Periodic breathing (PB) is a more encompassing term which includes CSA plus periods of hypoventilation rather than true apnoea. By definition, CSA should last at least three continuous cycles [3]. Although measured by the apnoea hypopnoea index (AHI) metric, an alternative and possibly better metric is the % of total sleep time (TST) in CSA. Although CSA has been described as unstable breathing by some—as opposed to stable or regular breathing‐ it is characterised by a pattern of picturesque continuous cyclic oscillatory breathing which is definitely rhythmic and predictable.

The periodic reduction in airflow seen with CSA, differs from obstructive sleep disordered breathing (which is due to upper airway narrowing) and disorders of non‐periodic hypoventilation seen with neuromuscular disorders and chest wall disease.

## 2 CSA Subtypes

The International Classification of Sleep Disorders [4] classifies CSA in adults into seven categories, the clinical features of which are summarised in Table 1.

1. Primary (or idiopathic) CSA
2. CSA with Cheyne–Stokes respiration (CSA‐CSR) (e.g., heart failure (HF))
3. CSA due to a medication (e.g., ticagrelor) or substance abuse (e.g., opioids)
4. CSA due to a medical disorder without Cheyne–Stokes (e.g., hypoventilation disorders related to motor neurone disease or obesity hypoventilation syndrome)
5. Treatment emergent CSA (TECSA)
6. CSA due to high altitude
7. Other
  - Renal failure
  - Pulmonary hypertension
  - Stroke

**TABLE 1:** Comparison of central sleep apnoea phenotypes.

	Prevalence	Gender	Cycle length	Morbidity	Mortality
High altitude	Universal > 4000 m altitude [5]	N/A	Short [6]	Fatigue, fragmented sleep, nocturnal awakenings	None—improves with descent or acclimatisation
Idiopathic	Rare [7]	Male predominant [7]	< 45 s [2, 7]	Fatigue, fragmented sleep, nocturnal awakenings	Nil established [8]
Opioid	24%–30% of chronic opioid users [9]	N/A	Variable [9]	Hypercapnia	May be increased [10]
Pulmonary HT	20%–30% of PAH patients, but generally mild [11]	—	< 45 s [6]	Fatigue, fragmented sleep, nocturnal awakenings	Nil established [8]
Heart failure	25%–40% of patients with HFrEF [12]	Male predominant [12]	> 45 s [2, 13]	Fatigue, fragmented sleep, nocturnal awakenings	Nil established [8]
Ch Kidney Disease	10% of patients with CKD [14]	Male predominant [14]	< 45 s [6]	Fatigue, fragmented sleep, nocturnal awakenings	Nil established [8]
Treatment Emergent CSA	1% of patients initiating CPAP for OSA [6]	Male predominant	< 45 s	Poor CPAP tolerance	Nil established [8]

Periodic limb movements, which often involve skeletal muscles of the chest wall, can give an appearance of PB, and thereby should be considered a differential diagnosis for CSA.

## 3 Common Physiology

Common to all forms of CSA is that there is a brief period of hyperventilation which drives PaCO_2_ below the apnoeic threshold (i.e., a level required to stimulate ventilation). The subsequent reduction in drive to ventilate (i.e., central apnoea) results in a rise in PaCO_2_ to a level that triggers further ventilation and ongoing oscillatory ventilation. A wide distance between eupnoeic and apnoeic CO_2_ levels provides a buffer to prevent CSA.

All forms of CSA occur mainly in transition between wakefulness and stage 1 and 2 non‐REM sleep when easily aroused (i.e., very sensitive, so called low arousal threshold). CSA is often triggered by an arousal.

Three background factors contribute to the development of CSA:

First is heightened chemosensitivity (also called controller gain) [15]. There is a dual relationship between rapidly responsive *peripheral* and slower responsive *central* chemoreceptors. Both are under sleep state control.

Anatomically, the peripheral chemosensors are the pea sized *carotid bodies* (CB) which sit close to the carotid artery bifurcation bilaterally. They are the most vascular structures in the body and made up of glomus cells. They are highly sensitive to changes in pH, CO_2_, O_2_, nitric oxide, hypoxia, glucose, haemoglobin and autonomic control [16]. The CB are innervated by an afferent (sensory) carotid sinus nerve (branch of the glossopharyngeal nerve) which carries signals to the nucleus tractus solitarius (a brain stem group of neurones which control autonomic nerve output) whereas the efferent modulatory nerves to the CB are carried by the glossopharyngeal nerve (parasympathetic) and superior cervical ganglion (sympathetic input). The activity of the peripheral chemoreflex is tested by the hypoxic ventilatory response.

The central chemosensor is positioned at the *medulla oblongata* with both inspiratory (pre Botzinger) and expiratory (Botzinger) groups of neurones. This area, also known as the ventral respiratory group (VRG), is sensitive to pH and CO_2_ (although significantly less than the carotid body). The central chemosensitivity is tested by the hypercapnic ventilatory response.

Second is the pulmonary effect on gas exchange (i.e., plant gain). Pulmonary changes are best demonstrated in HF characterised by pulmonary venous congestion, then interstitial oedema followed by alveolar oedema and thereafter (when lymphatics are overloaded) by pleural effusions [17]. Functionally, this is characterised by a restrictive ventilatory defect with impaired DLCO and increased dead space ventilation [17, 18]. As ~50% of the body's O_2_ storage is kept in the lungs, a reduction in lung volume or gas exchange will create greater swings in levels of blood oxygen and to a lesser extent CO_2_.

Third is the time delay between the lungs (the “plant”) and the chemosensors (CB and VRG, controller gain) leading to asynchrony between sensitivity and the output to breathe for which the term loop gain has become popular. Time delay is best exemplified in CSA‐CSR due to HF, the delay relates to greater sized mixing chamber (i.e., enlarged left ventricle) and reduced cardiac output [13].

## 4 Heart Failure and CSA

Advanced HF results in a characteristic type of CSA‐CSR: a waxing and waning pattern of ventilation which occurs during non‐REM 1 and 2 sleep, often triggered by a state change or arousal. Approximately 30–45 s of hyperventilation is followed by 30–45 s of apnoea without respiratory effort associated with hypocapnia and minimal hypoxemia. Typically, such patients have advanced HF of any cause (pump, valve or rhythm) with an elevated pulmonary capillary wedge pressure. Symptomatically patients often complain of insomnia and orthopnoea. Snoring is either absent or limited to the periods of hyperventilation when ventilation may change from nasal to oronasal.

With the advancement of HF, two physiological sequelae relevant to CSA‐CSR occur: (a) pulmonary capillary wedge pressure rises which leads to vagal afferent feedback and (b) heightened sympathetic nerve (SNA) [19]. Both lead to increased chemosensitivity (controller gain) and thereby hypocapnia. In addition, there are pulmonary changes (as outlined above) leading to plant gain. Given HF and reduced cardiac output, the triad of increased controller and plant gain with circulatory delay creates the optimal condition for CSA‐CSR.

Why CSA‐CSR occurs mainly in men, rather than women, remains unknown. It is possible female hormones stabilise ventilation. An alternative view is that women have reduced respiratory muscle strength and are unable to generate sufficient hyperventilation to trigger CSA.

Treatment Option #1: Treat the upstream cause namely HF:

It has long been established that interventions to treat HF improve associated CSR‐CSA. However historically, normalisation of cardiac function has been difficult to achieve. As the available options for treating HF have grown in number and efficacy, there has been increasing evidence showing that CSR‐CSA can be normalised by treating underlying heart failure.

1. Medications: Guideline‐based medical therapy (GBMT) has been shown to significantly increase LV function, and is first line therapy for most HFrEF patients [20]. Carvedilol [21], sacubitril‐valsartan [22] and SGLT2 inhibitors [23] have all been shown to reduce AHI.
2. Cardiac Resynchronisation Therapy (CRT): is an effective treatment for patients with HFrEF and left bundle branch block. CSR‐CSA has been shown to be prevalent in patients referred for CRT [24], and a review of five non‐randomised studies has shown consistent reductions in AHI and CAI post CRT [24, 25].
3. Aortic and mitral valve repair has been shown to significantly decrease AHI and CAI [17, 26], which occurred in concert with a reduction in pulmonary capillary wedge pressure.
4. Mechanical Circulatory Assistance with a left ventricular assistance device (LVAD) has been previously described in case reports [27, 28]. This is supported by a recent retrospective review demonstrating marked reductions in CAI post LVAD implantation in a cohort of 350 advanced HF patients [29].
5. Cardiac transplantation significantly improves haemodynamics [30]. In a population of 13 patients with HF and CSA, cardiac transplantation ameliorated CSA in 10 patients. Four patients did however acquire OSA [31]. The persistence of central events in some patients may reflect incomplete normalisation of cardiac haemodynamics and/or an incomplete reversal of the heightened central chemosensitivity observed in heart failure.
6. Electrophysiological ablation for atrial fibrillation in patients without HF has been shown to decrease AHI from 22 to 15 amongst those who maintained sinus rhythm [32]. The utility of ablation in treating CSA in patients with both HFrEF and AF has yet to be formally elucidated.
7. Cardiac Rehabilitation: Although there have been no polysomnographic studies pre‐ and post‐cardiac rehabilitation, it has been shown that exercise oscillatory ventilation (EOV) significantly improves. In a group of 52 HF patients undergoing cardiac rehabilitation, 37 experienced complete resolution of EOV compared to only 1 out of 44 patients in the control group [33]. EOV and CSR‐CSA commonly co‐exist and are likely to be due to similar pathologic mechanisms.
8. Home inotropic support: Similarly, EOV has been shown to be completely ameliorated in patients receiving home inotropes (milrinone) or cardiac transplantation. In a group of 32 patients with advanced heart failure, 5 were found to have EOV on baseline cardiopulmonary exercise test. All patients eventually normalised their breathing pattern—3 post milrinone and 2 post cardiac transplantation [34].

Treatment Option #2: Consider “Residual” CSA‐CSR post HF treatment as adaptive.

Although CSA‐CSR is associated with increased cardiovascular mortality, to date there is little evidence that CSA‐CSR (without OSA) is a direct cause of increased mortality independent on HF severity [35]. In the SERVE‐HF trial [36], in which 1325 adult patients with GBMT HF with predominantly CSA were randomised to adaptive servo‐ventilation (ASV) with pressure support or GBMT over a median of 31 months. The randomised to ASV had a greater all cause and cardiovascular mortality. Although the active group may have contributed to increased mortality, it is possible CSA‐CSR was protective against mortality. In the Sleep Heart Health Study, in which 6441 community dwellers aged > 40 years had home sleep studies and followed for an average 8.2 years, CSA was not associated with increased mortality [8].

The treatment of underlying HF is associated with proportional reductions in CSA‐CSR. In a perfect world, if there is complete normalisation of HF (and chemosensitivity and plant gain) then CSA‐CSR should resolve. The world is not perfect and residual CSA‐CSR often occurs leading clinicians to a dilemma whether to intervene or not.

One argument has been that residual CSA‐CSR hosts adaptive or compensatory mechanisms to assist HF, which can be summarised as follows:

1. During the hyperventilation period, there are large deep breaths which
  - increase in end expiratory lung volume (greater O_2_ stores)
  - assist stroke volume
  - attenuate the heightened SNA
2. Mechanically a period of increased work (hyperventilation) followed by a period of rest (central apnoea), allows overall minute ventilation to become more efficient, and thereby the respiratory pump muscles to require a lower proportion of the (reduced) cardiac output
3. The prevailing “respiratory alkalosis”, compared with acidosis, will be a favourable environment for cardiac function under potentially hypoxic conditions
4. The apnoea in CSA‐CSR (when measured by exhaled capnography) is often a prolonged expiration [37]. In some cases, this expiration slows midway through an apnoea, at which time the upper airway passively closes. If expiration continues (elastic recoil) with a closed upper airway, a small amount of PEEP (~5 cm H_2_O) can result. This small amount of PEEP may be sufficient to prevent alveolar collapse.

These detailed descriptions of CSA‐CSR in HF have been hampered by measurement techniques (absence of measuring ventilation and respiratory effort accurately) and the ubiquitous reliance on a simple and sometimes misleading metric, the AHI.

Treatment Option #3: Directly treat downstream CSA‐CSR .

Alternatively, traditional tools used by respiratory & sleep clinicians to treat the CSA‐CSR directly have been trialled.

1. CPAP is commonly used in patients with CSR‐CSA. This is based upon the physiological advantages of CPAP in acute pulmonary oedema: namely *pulmonary* (increasing lung volume, preventing alveolar collapse, assisting inspiratory activity and bronchodilation) and *cardiac effects* (reduction in left ventricular transmural pressure) whilst preventing pharyngeal collapse. In patients with concomitant OSA, CPAP has been shown to improve LV function [38]. However, the widely cited multicentred CANPAP trial of CPAP in CSR‐CSA, improvements in AHI, SpO_2_, PaCO_2_, serum catecholamines, LVEF and 6‐min walk distance were observed with CPAP, but did not demonstrate a transplant‐free survival advantage [39].
2. Adaptive Servo‐Ventilation (ASV) is a specific type of bilevel positive airway pressure support designed to stabilise breathing pattern in CSA. ASV utilises a fixed expiratory pressure, variable pressure support and backup rate. Although ASV has been shown to significantly reduce AHI, the largest randomised trial to date (SERVE‐HF) demonstrated a significantly increased risk of cardiovascular death and all‐cause mortality amongst patients with HFrEF on ASV [36]. The more recent ADVENT‐HF trial attempted to challenge this finding using an alternative, peak flow based ASV algorithm, across a broader range of HF patients with CSA and OSA but was likely underpowered due to the COVID‐19 pandemic halting recruitment and device supply issues due to the Phillips pump recall [40].
3. Phrenic nerve stimulation is a relatively novel treatment for CSA, which uses a generator and transvenous lead to stimulate unilateral diaphragm contraction during the central apnoeas. The largest studies of phrenic nerve stimulation in CSA were the Pivotal study and follow‐up Post‐Approval study, which enrolled patients with CSA of any cause except opiates. Phrenic nerve stimulation was highly effective in reducing AHI [41]. Epworth sleepiness scale (ESS) was also reduced, although baseline ESS was in the ‘high normal’ rather than abnormal range. Despite the reduction in AHI, polysomnographic data demonstrated persistently minimal proportions of N3 sleep at 5‐year follow‐up (1% of TST), suggesting that phrenic nerve stimulation may independently influence sleep architecture. To our knowledge, outside of Japan, phrenic nerve stimulation is not available in the Asia Pacific region, further limiting feasible access for patients.
4. Oxygen: Several non‐randomised studies have evaluated the use of nocturnal oxygen, which results in hyperoxia and consequently reduced controller gain. Although effective in reducing AHI [42, 43, 44], hyperoxia has been associated with adverse sequelae including reduced cardiac output [45] and coronary artery vasoconstriction [46]. International guidelines do not currently recommend long‐term oxygen therapy for normoxic patients with HF [47].

## 5 Drug Induced CSA

Opioids

Opioids are commonly used and are a common cause of periodic CSA. Correa et al. conducted a review of eight studies comprising 560 adult subjects with chronic pain & addiction (male:female ratio 40:60), methadone was the most common opioid [48]. Seventy percent of subjects had an AHI > 5. An estimated 24% (14%–60%) of subjects had CSA. A minority had ataxic breathing or OSA. All studies indicated a low baseline SpO_2_ and mildly elevated PCO_2_—the cause of which may relate to mild prevailing hypoventilation and/or VQ mismatch. The studies were confounded by concomitant benzodiazepine or hypnotic use. The risk factors for developing CSA appeared to be a morphine equivalent daily dosage of > 200 mg and a low‐normal BMI.

Although there is no long‐term morbidity or mortality data available for this group, an important clinical message, in the perioperative environment, is that acute opioids can disable the normal protective arousal response to hypoxia.

The pathogenesis of SDB in chronic opioid use relates to 4 locations of Mu receptors in the VRG and CB, plus bulbospinal pre‐motor neurones (respiratory pump muscles) and hypoglossal motor nucleus (upper airway patency) [49].

Given the prevalence and severity of opioid‐induced CSA correlates with daily equivalent morphine dose [9], hence reduction of opioid dose is the preferred treatment when feasible. In patients in whom dose reduction is not feasible, bilevel NIV with a backup rate or ASV can be considered [50, 51]. CPAP has been shown to be ineffective in this cohort [52].

Ticagrelor

Recent reports arose from a comparative trial of anticoagulants (ticagrelor vs. prasugrel) in patients with acute coronary syndrome [53]. The group treated with ticagrelor had a 3‐fold greater AHI (31 vs. 10) and greater HCVR compared with the prasugrel group without impairing the hypoxic burden.

## 6 Idiopathic CSA

This disorder is characterised by short cycle length (30–45 s) CSA for which no cause is found. A thorough cardiac and neurological assessment is recommended with a drug screen (opioids) plus exclusion of unusual endocrine causes of hyperventilation such as thyrotoxicosis, hyperglycaemia and phaeochromocytoma. Exclusion of brain stem pathology should also be considered.

Several interventions have been trialled in idiopathic CSA however the vast majority of these trials have been non‐randomised and have not demonstrated a prognostic benefit.

1. Positional therapy: Lateral rather than supine sleep has been shown to significantly decrease AHI across both NREM and REM sleep in patients with CSR‐CSA [54]. This may be due to increased functional residual capacity, reduced pharyngeal collapse and/or reduced pulmonary congestion.
2. Medications
  - Several non‐randomised studies have demonstrated reductions in AHI and CAI with acetazolamide [55, 56] or theophylline [57].
  - GABAergic hypnotics have also been trialled in several small studies, demonstrating reduction in AHI likely due to an increased arousal threshold [58, 59, 60]. However, no studies have demonstrated a symptomatic or quality of life benefit. It should also be noted that GABAergic medications, including benzodiazepines, may cause CSA in rare cases [61].
3. CO_2_: Raising arterial CO_2_ may reduce central apnoeas by widening the CO_2_ reserve. Both administration of exogenous CO_2_ and addition of anatomic dead space have been shown to reduce AHI [62]. However, it has been demonstrated that raising arterial CO_2_ may independently cause cortical arousals and increase sympathetic tone, which may counteract the improvement in sleep consolidation resulting from treating central apnoeas [63].
4. CPAP has been shown to improve central apnoeas in some ICSA cases [64, 65].

## 7 High Altitude Induced CSA

CSA is universal in lowlanders sojourning above altitudes of 3000 metres [66]. At 3000 m, CSA occurs ~40% of total sleep time (TST) increasing to 100% at 8000 m. Similar concerns have been raised that the CSA at altitude may be pathological leading to sympathovagal imbalance and the development of a pro‐inflammatory maladaptive state as has been considered with CSA sea level [1]. Moreover, CSA at altitude was thought to cause hyperventilation, and thus increased work of breathing (WOB) and increased oxygen demands.

Two important studies recently published refute the maladaptive theory. Both WOB and alveolar gas exchange were measured during simulated 3500‐m altitude induced CSA in a group of 20 healthy males [67, 68].

The respiratory pattern in periodic, compared with regular breathing, was characterised by a slower respiratory rate (10.6 v 16.8), a greater tidal volume (0.815 vs. 0.633 L) with an overall lower minute volume of ventilation whilst maintaining alveolar ventilation and mean SpO_2_. The authors also reported that CSA provided a 17% reduction in WOB as measured by the inspiratory pressure time product compared with RB without impairing gas exchange [68]. Thus, CSA at altitude is not only adaptive, but also offers a physiological advantage in terms of reduced WOB and by inference oxygen consumption without compromising gas exchange. The reported greater duration of CSA (as a % of TST) with the greater altitude would support this concept [68].

Acetazolamide is commonly used in order to create a mild metabolic acidosis, resulting in hyperventilation and lowering the apnoeic threshold. This accelerates the natural process of acclimatisation that usually occurs over several days. Acetazolamide prophylaxis has been shown to improve symptoms of altitude sickness and polysomnographic data, both in all‐comers and in patients with OSA [69, 70, 71]. However as noted previously, pharmacotherapy may not always be required, particularly for individuals who are asymptomatic. Descent, where feasible, remains the most effective treatment for high‐altitude related illness, including CSA [72].

## 8 Treatment Emergent CSA

In 5% of standard OSA patients who initiate CPAP, treatment emergent CSA (TECSA) will be observed. However, the majority resolve within 30 days. If persistent, it is wise to check PAP settings (e.g., ensure set pressure is not too high—noting the Hering–Breuer effect of hyperinflated lungs). It is this author's approach to reassess CPAP pressure in ambulatory patients to a pressure in cmH_2_O equivalent to 10% body weight (in kg). Also ensure that underlying periodic limb movements or cardiac impairment (pump, valve or rhythm) have been excluded.

A second form of TECSA occurs when a patient has been using CPAP for some months or years, and the download shows a stepwise increase in AHI, often central in type. Once mask leak, other factors that might indicate inadequate pressure settings and periodic limb movements have been excluded, atrial fibrillation is often causal [73].

The use of acetazolamide [74] and or dead‐space ventilation (through CPAP) [75] in refractory TECSA has been previously described, however these practices are not yet backed up by substantial trial evidence.

## 9 Renal Failure and CSA

In patients with advanced renal failure, CSA can be seen. The precise mechanisms responsible are not clear however excess fluid (elevation of PCWP), sympathovagal imbalance, periodic limb movements are all possible culprits. Treatment of the underlying renal failure and its complications (anaemia, excessive fluid and electrolyte disturbance) should be considered before trialling therapy directed at the CSA specifically (i.e., PAP, acetazolamide, oxygen). A hallmark paper described the utility of frequent nocturnal dialysis on reducing CSA [76].

## 10 Pulmonary hypertension and CSA

Both obstructive and central sleep apnoea are more prevalent in pulmonary hypertension compared to the general population [77]. By definition, these patients have preserved left ventricular function, however in advanced or decompensated disease, cardiac output is reduced due to right ventricular failure, leading to increased mixing gain and contributing to central sleep apnoea [78].

Nocturnal hypoxaemia is common in this group, which is generally a result of the underlying pulmonary vascular dysfunction rather than respiratory events [79].

## 11 Post Stroke and CSA

Sleep disordered breathing is highly prevalent in stroke patients, but may be present in several different forms [80, 81]. Stroke risk is elevated in severe obstructive sleep apnoea, and hence many patients are found to have significant OSA after the sentinel event of a stroke [82]. Another group of patients are those with subclinical or established cardiac failure, who share many risk factors for stroke and may have pre‐existing central sleep apnoea. Patients may also develop central sleep apnoea due to cerebral infarction post‐stroke.

We recommend that patients with post‐stroke sleep disordered breathing undergo a comprehensive assessment to exclude cardiac failure. It may also be appropriate to screen for hypoventilation (with a serum bicarbonate ± pCO_2_).

OSA should certainly be treated where possible post stroke, which has been shown to improve stroke outcomes in selected populations [83]. For patients with significant CSA, a CPAP trial is recommended, ideally with an in‐lab titration.

## 12 Conclusion

Periodic CSA can occur in a number of common circumstances: HF, altitude and opiates. Unfortunately, standardised questionnaires, such as ESS or StopBang, are not designed to identify such patients. Measurement technique (simplified vs. detailed with markers of ventilation and respiratory effort) and scoring (discerning central from obstructive hypopnoea) have been a challenge for the field.

Simply measuring cycle length should assist in determining a long CL (e.g., > 45 s in HF) from a short CL (idiopathic or opioid induced).

Greater mortality due to the CSA‐CSR (rather than the HF itself) has never been shown. In the SERVE‐HF trial, ASV was associated with increased all‐cause mortality. The presence of CSA‐CSR was associated with an improved survival. In the large SHHS, the presence of CSA did not portend an increased mortality over 8.2 years [8].

The weight of evidence to treat the cause (e.g., HF) rather than the end result (CSA‐CSR) is increasing [8, 36, 67].

## Funding

The authors have nothing to report.

## Disclosure

The authors have nothing to disclose.

## Conflicts of Interest

The authors declare no conflicts of interest.
